# Supplementary material for: Androgen-Regulated microRNAs (AndroMiRs) as Novel Players in Adipogenesis
Source: Int J Mol Sci. 2019 Nov 16;20(22):5767. doi: 10.3390/ijms20225767 (PMC6888160; doi:10.3390/ijms20225767)
Supplement: Supplementary file 1 [file ijms-20-05767-s001.pdf]

Table S1. Adipogenesis-regulating microRNAs in mouse.

micro RNA	Effect on adipogenesis	Cell system	Target gene/signaling pathway	Reference
miR-10b-5p	i	3T3L1	Apol6	Tan et al., 2019
miR-17-5p	p	3T3L1	TCF7L2	Tian et al., 2017
miR-17-92	p	3T3L1	Rb2/p130	Wang et al., 2008
miR-20a	p	3T3L1	KDM6B, TGTBR2	Zhou et al., 2015
miR-23a	i	3T3L1		Shen et al., 2016
miR-24	p	3T3L1	MAPK7	Jin et al., 2016
miR-25	i	3T3L1	KLF4; CEPBa	Liang et al., 2015
mir-26b	p	3T3L1	PTEN	Li et al., 2017
miR-27a	i	3T3L1	PPARg	Kim et al., 2010
mir-29a/b/c	i	3T3L1	DNMT3A	Zhu et al., 2017
miR-103	p	3T3L1	MEF2D, TNFa	Xie et al., 2009, Li et al., 2014
miR-103	p	3T3L1	Thy1 (CD90)	Woeller et al., 2017
miR-124	p	3T3L1	DLX5	Qadir et al., 2013
miR-128-3p	i	3T3-L1	PPARγ, Sertad2	Chen at al., 2018
miR-135a-5p	i	3T3L1	Apc	Chen et al., 2014
miR-139-5p	i	3T3L1	Notch1, IRS1	Mi et al., 2015
miR-140-5p	p	3T3L1; ST2	Tgfbr1	Zhang et al., 2015
miR-143	p	3T3L1	PTN, TNFa	Yi et al., 2011, Xie et al., 2009
miR-143a-3p	i	3T3L1	MAPK7	Zhang et al., 2018
miR-144-3p	i	3T3L1	Klf3, CtBP2	Shen et al., 2018
miR-146b	i	3T3L1	SIRT1	Ahn et al., 2013
miR-148a	p	3T3L1	Wnt10b	Cho et al., 2016
miR-148a-3p	p	ST2	Kdm6b	Tian et al., 2017
miR-155	i	3T3L1	C/EBPb, CREB	Lin et al., 2011
miR-181a-5p	p	3T3L1	Smad7; Tcf7l2	Ouyang et al., 2016
miR-183	i	3T3L1	LRP6	Chen et al., 2014
miR-185	i	3T3L1	SREBP1	Ning et al., 2017
miR-188	p	BMSC	HDAC9, RICTOR	Li et al., 2015
miR-191	i	3T3L1	CEBPb	Ji et al., 2014
mir-199a-3p	p	3T3L1	SCD	Tan et al., 2017
miR-200b	p	3T3L1	KLF4	Shen et al., 2018
miR-204-5p	p/i	3T3L1	Bcl-2	Du et al., 2018
miR-206-3p	i	3T3L1	c-Met	Tang et al., 2017
mir-210	p	3T3L1	Tcf7l2	Qin et al., 2010
miR-215	i	3T3L1	FNDC3B, CTNNBIP1	Peng et al., 2016
miR-223	p	C3H10T1/2; ST2	Fgfr2	Guan et al., 2015
miR-224-3p	p	3T3L1	EGR2, ACSL4	Peng et al., 2013
miR-302a	i	3T3L1	PPARg	Jeong et al., 2014
miR-344	i	3T3L1	GSK3b	Chen et al., 2014
miR-375	p	3T3L1	ERK-PPARg2-AP2	Ling et al., 2011
miR-378a-3p	p	3T3L1	MAPK1	Huang et al., 2015
miR-3963	p	mouse MSC	C/EBPα and PPARγ	Liu et al., 2018
miR-425-5p	p/i	3T3L1	Mapk14	Qi et al., 2019
miR-483-5p	i	3T3L1		Zhang et al., 2016
miR-540	i	3T3L1	PPARg	Chen et al., 2015
miR-709	i	3T3L1	GSK3b	Chen et al., 2014

Table S2. Adipogenesis-regulating microRNAs in other species.

micro RNA	Effect an adipogenesis	Cell system	Target gene/signaling pathway	Reference

miR-15a/b	p	porcine adipocytes	FoxO1	Dong et al., 2014
miR-27	p	porcine adipocytes		Wang et al., 2011
miR-33b	i	porcine adipocytes	EBF1	Tamiguchi et al., 2014
miR-125a-5p	p/i	porcine preadipocytes	Klf13, ELOVL6	Du et al., 2018
miR-127	i/p	porcine adipocytes	MAPK4,HOXC6	Gao et al., 2019
miR-130b	i	GMEC	PGC1a	Chen et al., 2015
miR-145	i	porcine adipocytes	IRS1	Guo et al., 2012
miR-148a-3p	p	rabbit preadipocytes	PPARγ, C/EBPα, and FABP4, PTEN	He at al., 2018
miR-181a	p	porcine adipocytes	TNFα	Li et al., 2013
miR-181a	p	porcine preadipocytes	TGFBR1	Zhang et al., 2019
miR-183	i	hircine preadipocytes	PPARγ, C/EBPα, SREBP-1c, FAS, and ACC	Zhao et al., 2018
miR-199a-5p	p	porcine adipocytes	Cav-1	Shi et al., 2014
miR-429	i	porcine	KLF9; p27	Peng et al., 2016

Table S3. microRNAs regulating AR expression.

microRNA	tissue	target gene/ signaling pathway	reference
let-7c	PCa	AR	Nadinity et al., 2012
miR-1	PCa	AR	Stope et al., 2013
miR-1	PCa	AR	Siu et al., 2016
miR-30c, miR-103a	PCa	AR	Chen et al., 2019
miR-1207-3p	PCa	AR	Das et al., 2016
mir-124	bladder cancer	AR	Xiaong et al., 2017
miR-124a	TCC	AR	Stanley et al., 2012
miR-124	PCa	AR	Shi et al., 2013
miR-124	PCa	AR, EZH2, SRC	Shi et al., 2015
miR-130a	mouse Sertoli cells	AR	Li et al., 2018
miR-130b	PCa	AR	Cammostraco et al., 2017
miR-135b	BCa/PCa	AR	Aakula et al., 2015
miR-141-3p	PCa	AR	Wang et al., 2015
miR-145	PCa	AR	Larne et al., 2015
miR-185	PCa	AR	Qu et al., 2013
miR-185	PCa	AR	Liu et al., 2015
miR-204	PCa	AR	Todorova et al., 2017
miR-205	Caval bodies	AR	Wen et al., 2019
miR-205	PCa	AR	Hagman et al., 2013
miR-298	muscle	AR	Pourshafie et al., 2016
miR-301a	Pca/pre-adipocytes	AR	Xie et al., 2015
miR-30b/d	PCa	AR	Kumar et al., 2016
miR-3148	cardiomyocytes	AR	Miao et al., 2017
miR-3162-5p	PCa	AR, KLK2-4	Martin et al., 2019
miR-320b	PCa	AR, MALAT-1	Deu et al., 2019
miR-320b	PCa	AR	Sato et al., 2016
miR-331	PCa	AR, ERBB2	Epis et al., 2009
miR-34	PCa	AR	Shiina et al., 2017
miR-34, miR-449	PCa	AR	Lin et al., 2017
miR-346, miR-361-3p, miR-197	PCa	AR	Fletcher et al., 2019
miR-34a	PCa	AR	Kashat et al., 2012
miR-367	HCC	AR, MDM2	Xu et al., 2016
miR-34a, miR-371	PCa	AR	Leite et al., 2015
miR-381	PCa	AR	Rui et al., 2019
miR-449a	bladder cancer	AR	Guo et al., 2018
miR-449a	PCa	AR	Zheng et al., 2015
miR-488*	PCa	AR	Sikand et al., 2011
miR-488*	PCa	AR	Ebron et al., 2016
miR-541	PCa	AR	Hu et al., 2015
miR-582, miR-637	lung cancer	AR	Jin et al., 2017
miR-762	boar sperm cells	AR	Ma et al., 2016
